# Effect of Water Stress during Grain Filling on Yield, Quality and Physiological Traits of Illpa and Rainbow Quinoa (*Chenopodium quinoa* Willd.) Cultivars

**DOI:** 10.3390/plants8060173

**Published:** 2019-06-14

**Authors:** Angie L. Gámez, David Soba, Ángel M. Zamarreño, José M. García-Mina, Iker Aranjuelo, Fermín Morales

**Affiliations:** 1 Instituto de Agrobiotecnología – CSIC, Avenida de Pamplona 123, 31192 Mutilva, Spain; angie.gamez@unavarra.es (A.L.G.) david.soba@unavarra.es (D.S.) iker.aranjuelo@csic.es (I.A.); 2 Universidad de Navarra, Facultades de Ciencias y Farmacia y Nutrición, Grupo de Biología y Química Agrícola (Departamento de Biología Ambiental), c/Irunlarrea 1, 31008, Pamplona, Spain; angelmarizama@unav.es (Á.M.Z.); jgmina@unav.es (J.M.G.-M.); 3 Dpto. Nutrición Vegetal, Estación Experimental de Aula Dei (EEAD), CSIC, Apdo. 13034, 50080 Zaragoza, Spain

**Keywords:** leaf abscisic acid, quinoa yield and quality, quinoa varieties, stomatal closure, water deficit

## Abstract

The total area under quinoa (*Chenopodium quinoa* Willd.) cultivation and the consumption of its grain have increased in recent years because of its nutritional properties and ability to grow under adverse conditions, such as drought. Climate change scenarios predict extended periods of drought and this has emphasized the need for new crops that are tolerant to these conditions. The main goal of this work was to evaluate crop yield and quality parameters and to characterize the physiology of two varieties of quinoa grown under water deficit in greenhouse conditions. Two varieties of quinoa from the Chilean coast (Rainbow) and altiplano (Illpa) were used, grown under full irrigation or two different levels of water deficit applied during the grain filling period. There were no marked differences in yield and quality parameters between treatments, but the root biomass was higher in plants grown under severe water deficit conditions compared to control. Photosynthesis, transpiration and stomatal conductance decreased with increased water stress in both cultivars, but the coastal variety showed higher water use efficiency and less discrimination of ^13^C under water deficit. This response was associated with greater root development and a better stomatal opening adjustment, especially in the case of Rainbow. The capacity of Rainbow to increase its osmoregulant content (compounds such as proline, glutamine, glutamate, K and Na) could enable a potential osmotic adjustment in this variety. Moreover, the lower stomatal opening and transpiration rates were also associated with higher leaf ABA concentration values detected in Rainbow. We found negative logarithmic relationships between stomatal conductance and leaf ABA concentration in both varieties, with significant R^2^ values of 0.50 and 0.22 in Rainbow and Illpa, respectively. These moderate-to-medium values suggest that, in addition to ABA signaling, other causes for stomatal closure in quinoa under drought such as hydraulic regulation may play a role. In conclusion, this work showed that two quinoa cultivars use different strategies in the face of water deficit stress, and these prevent decreases in grain yield and quality under drought conditions.

## 1. Introduction

The grain of quinoa (*Chenopodium quinoa* Willd.) is a pseudo-cereal with attractive nutritional proprieties, and this attribute has greatly increased its consumption in recent years [1]. Quinoa contains unsaturated fatty acids, antioxidants, and essential amino acids, and it is rich in Fe, Mg, fiber, and vitamins while containing high levels of gluten-free protein [2,3]. Because the quinoa plant shows high phenotypic and genetic variability, interest in this crop has also increased globally. This diversity allows it to grow under severe environmental conditions [4], such as dry and alkaline soils [5]. In the context of climate change, predictions foresee that precipitation will decline and drought periods will extend [6], with a strong impact on agriculture. The search for new tolerant crops to face these adverse conditions is thus important to generate adaptation strategies in agriculture [7]. Breeding programs should be based on analyses of crop physiological and agronomical characteristics to develop desirable cultivars of quinoa adapted to different environmental conditions [8]. In this sense, quinoa crops may be an alternative for semiarid and arid areas where other crops produce poor quality grain or are unable to grow.

Several studies on quinoa have been carried out to evaluate its morphological and physiological responses under water deficit [9,10,11,12,13]. Among them, quinoa shows high root plasticity [14] as well as leaf adaptations such as bladders with hygroscopic calcium oxalate crystals [15] and fast leaf abscission [9]. In response to water deficit, the physiological parameters of photosynthesis, stomatal conductance and transpiration decreased, but after 10 days of growth under stress these parameters remained stable with high water use efficiency [9,10]. Due to these characteristics, a number of authors have classified the quinoa plant as being tolerant of drought [9,10]. Additionally, some studies have found increases in the inorganic ions Na, Ca and Mg in the plant’s tissues under water deficit, maintaining the leaf cell turgor pressure in order to contribute to the osmotic adjustment under this condition [12]. 

Drought stress when it occurs at the grain filling stage can reduce plant yield substantially. Decreases close to 79–81% in maize [16] and 40% in durum wheat [17] have been reported in the literature. Other drought effects observed have been a shortened grain filling period in triticale genotypes [18] and an acceleration of the remobilization of carbon reserves to grain in rice [19]. With regard to grain quality, an increase in grain proteins was reported in durum wheat when water stress occurred during the grain filling stage [20]. In quinoa plants, no differences have been found in protein content (mainly albumins) under water stress [21]. However, little attention has been paid to the effect of water deficit on quinoa crop yields and quality with low water availability at the grain filling stage. 

Furthermore, at a molecular level, transcriptional studies in quinoa have shown a down-regulation of ABA-pathway biosynthesis genes in response to drought stress, and this might indicate that an ABA-independent mechanism acts in response to water deficit [22]. According to Jacobsen et al. [9], there was an apparent lack of root-sourced ABA regulation in quinoa under water deficit, which led these authors to suggest involvement of another chemical substance or a hydraulic regulation mechanism.

Taking into account all these factors, the main objective of this work was to evaluate the effect of water stress during the grain filling stage on crop yield and grain quality, to characterize the associated physiological changes and metabolite concentrations, and to identify possible osmotic regulators in two cultivars of quinoa from two different geographical regions grown under water deficit in greenhouse conditions. One of the strengths of the present work lies in the assessment of effects of water deficit in (i) a poorly investigated crop, quinoa; (ii) during a critical phenological period, grain filling; and (iii) linking plant physiology with grain yield and quality.

## 2. Results

### 2.1. Crop Yield, Grain Quality and Root Biomass under Water Stress

In this study, the two cultivars of quinoa showed differences in phenology throughout the whole life cycle. The coastal cultivar Rainbow grew for 120 days from sowing to harvest, whereas the altiplano cultivar Illpa grew for 240 days. Treatment applications were carried out 86 days after sowing (DAS) for Rainbow and 111 DAS for Illpa, when plants reached the beginning of the grain filling stage. No significant differences were detected in the grain yield between well-watered and water-stressed plants, irrespective of the cultivar analyzed, although Rainbow showed much higher grain yield than Illpa. With regard to thousand-grain weight, there was no difference in Rainbow, but in Illpa this trait decreased with the intensity of water stress (Table 1). 

**Figure 1:**
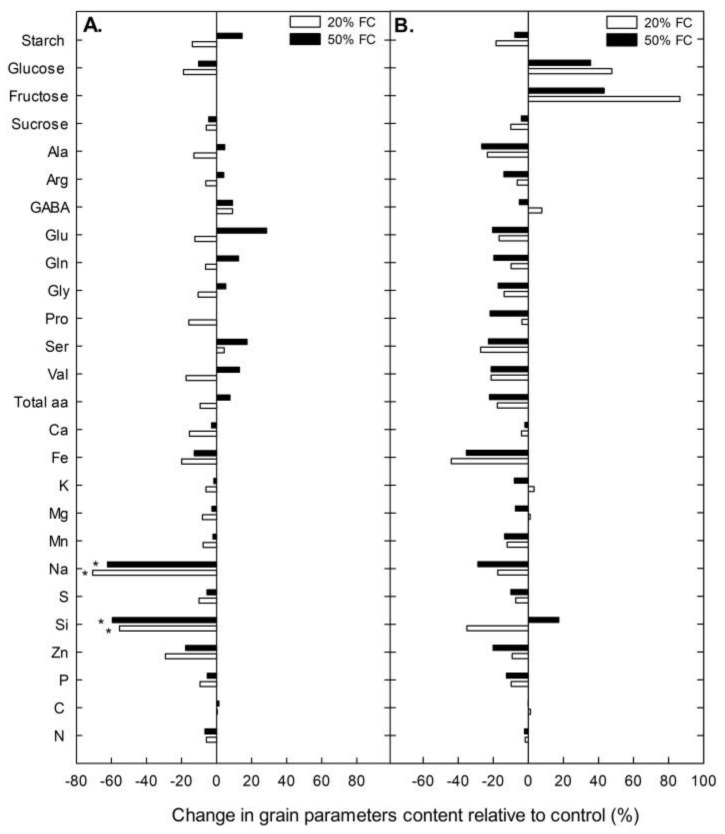
Change in grain quality parameters relative to control (%) in the Rainbow (**A**) and Illpa (**B**) cultivars grown under an irrigation regime of 100% substrate holding full capacity (FC), 50% FC and 20% FC. The asterisks indicate significant differences with p < 0.05 of the treatment relative to control (*n* = 7–10).

**Figure 2:**
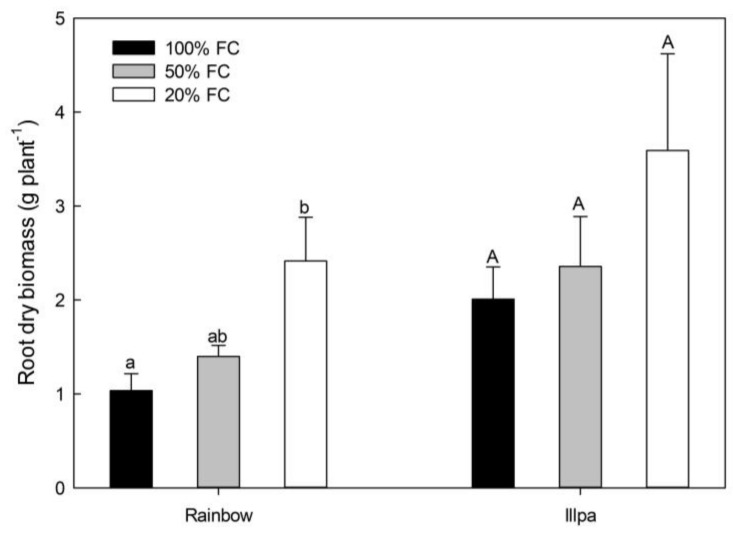
Root dry biomass (g plant^−1^) in the coastal (Rainbow) and altiplano (Illpa) cultivars grown under irrigation regimes of 100% substrate holding full capacity (FC), 50% FC or 20% FC. The same letters indicate no significant differences between treatments for a given cultivar (*p* < 0.05). Mean ± SE (*n* = 3–5).

Surprisingly, no significant differences were found among treatments in most of the grain quality variables in both cultivars (Supplementary 1) (although plants under water stress tended to have slightly lower values compared to the well-watered ones). The variables with significant differences (decreases in Na and Si in Rainbow) are shown in Figure 1. Root dry biomass increased under severe water stress conditions in both cultivars, although in Illpa the difference did not reach statistical significance due to the variability found in the 20% FC treatment (Figure 2).

### 2.2. Physiological Response to Water Stress

**Figure 3:**
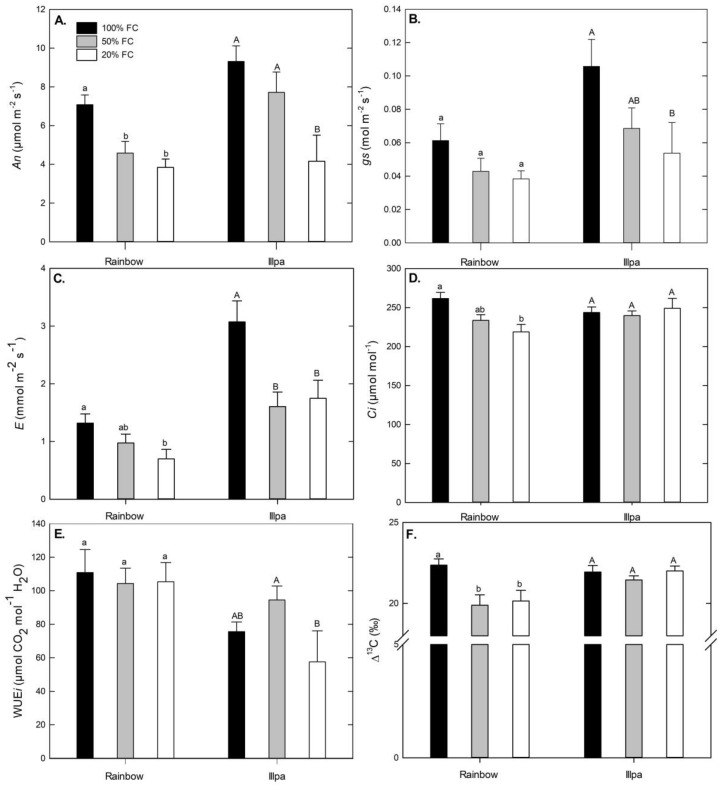
(**A**) Leaf photosynthetic rate (An, µmol m^−2^ s^−1^); (**B**) Stomatal conductance (gs, mol m^−2^ s^−1^); (**C**) Transpiration (E, mmol m^−2^ s^−1^); (**D**) Sub-stomatal CO_2_ concentration (Ci, µmol mol^−1^); (**E**) Intrinsic water use efficiency (WUEi = An/gs, µmol CO_2_ mol^−1^ H_2_O) and (**F**) Carbon isotope discrimination (Δ^13^C, ‰) in leaves of two quinoa cultivars (Rainbow and Illpa) grown under irrigation regimes of 100% substrate holding full capacity (FC), 50% FC or 20% FC. The same letters indicate no significant differences between treatments for a given cultivar (*p* < 0.05). Mean ± SE (*n* = 8–10).

Under full irrigation, Illpa had higher net photosynthesis (*An*) than Rainbow, possibly related to the higher stomatal conductance (*gs*) in the former than in the latter (Figure 3A,B). Net photosynthesis (*An*), stomatal conductance (*gs*) and transpiration (*E*) decreased in plants grown under water stress conditions, with significant differences in both cultivars except for *gs* in Rainbow (Figure 3A–C). Sub-stomatal CO_2_ concentration was significantly lower under severe water stress in Rainbow, but no differences among treatments were found in Illpa (Figure 3D). No differences were found in intrinsic water use efficiency (WUE*i* = *An*/*gs* ratio) in either cultivar when well-watered and water-stressed plants were compared, but interestingly Rainbow had higher WUE*i* than Illpa (Figure 3E). The ^13^C discrimination (Δ^13^C) was lower under moderate and severe water stress in Rainbow, whereas no differences were observed among treatments in Illpa (Figure 3F). 

### 2.3. Leaf Carbohydrates, Amino Acids and Mineral Concentration under Water Stress

**Figure 4:**
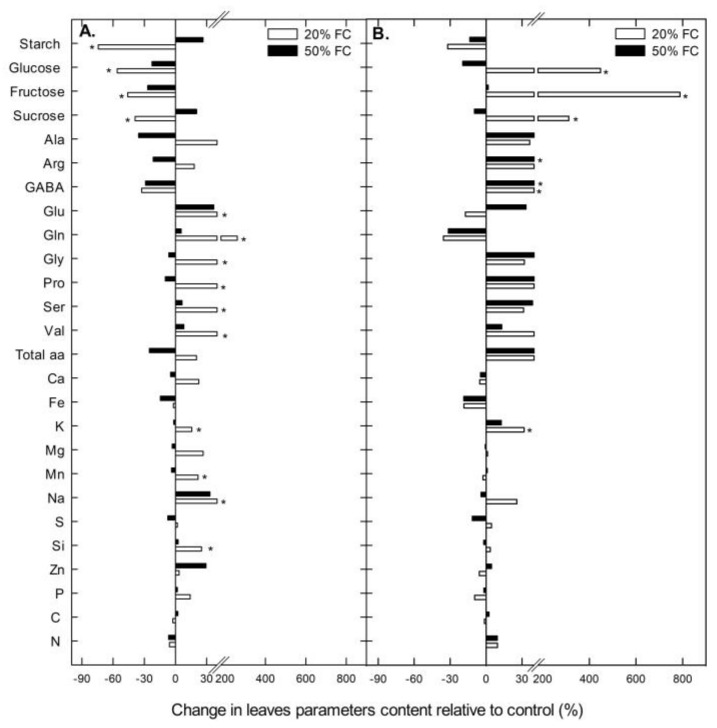
Change in carbohydrates, amino acids and mineral contents relative to control (%) in leaves of two quinoa cultivars (Rainbow – **A** and Illpa – **B**) grown under irrigation regimes of 100% substrate holding full capacity (FC), 50% FC or 20% FC. The asterisks indicate significant differences with *p* < 0.05 of treatment relative to control (*n* = 7–10).

When leaf carbohydrates, amino acids and mineral concentrations were analyzed in Rainbow, significant differences with respect to the controls were only found in plants grown under severe (20% FC) water stress. In Illpa, however, very few differences were found in plants grown under moderate and severe water stress when compared to the well-watered ones. For instance, the leaf starch concentration decreased significantly under severe water conditions in Rainbow but not in Illpa. Also, the soluble sugars (glucose, fructose and sucrose) decreased in Rainbow with water stress but, contrarily, in Illpa these sugars strongly increased under severe water stress (Figure 4). Values are shown in Supplementary 2. 

On the other hand, the water deficit caused an increase or decrease in leaf amino acid concentrations, although only the increases reached statistical significance (Figure 4). In Rainbow, glutamate, glutamine, glycine, proline, serine and valine increased significantly under severe water stress. In Illpa, two different amino acids, arginine and GABA, were increased (Figure 4).

In most of the leaf mineral concentrations, there were no significant differences among treatments in either cultivar, except for the strong Na increase in Rainbow (see Supplementary 2 for other minor increases) and K increase in Illpa in plants grown under severe water deficit (Figure 4).

### 2.4. Relative Rubisco Concentration under Water Stress

**Figure 5:**
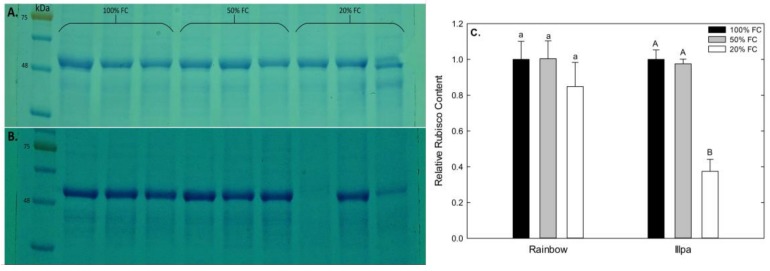
Rubisco detected by Western Blot in (**A**) Rainbow and (**B**) Illpa cultivar grown under irrigation regimes of 100% substrate holding full capacity (FC), 50% FC or 20% FC. (**C**) Relative Rubisco content in both cultivars. The same letters indicate no significant differences between treatments for a given cultivar (*p* < 0.05). Mean ± SE (*n* = 3).

Gels representative of the Rubisco analyses made in this study are shown in Figure 5A,B. The relative Rubisco concentration tended to be lower in plants grown under severe water stress in both cultivars. However, only Illpa showed a strong decline in the 20% FC treatment with respect to the well-watered control (Figure 5). 

### 2.5. Leaf ABA Concentration under Water Stress

**Figure 6:**
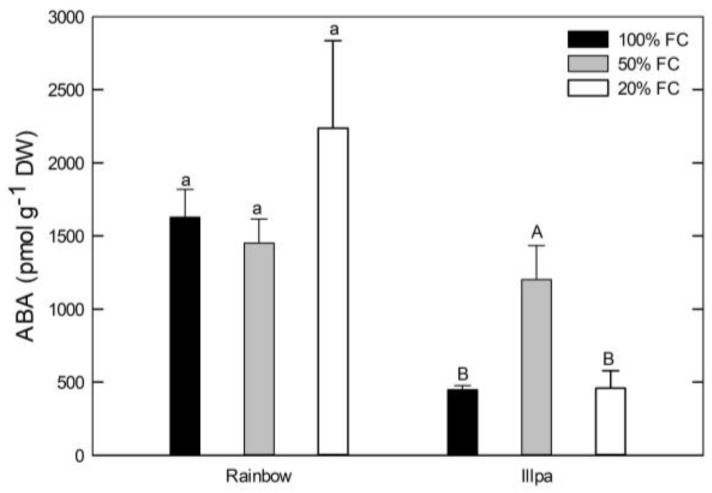
Leaf ABA concentration (pmol g^−1^ DW) in two quinoa cultivars (Rainbow and Illpa) grown under irrigation regimes of 100% substrate holding full capacity (FC), 50% FC or 20% FC. The same letters indicate no significant differences among treatments for a given cultivar (*p* < 0.05). Mean ± SE (*n* = 3–5).

With regard to leaf ABA, no significant differences among treatments were found in Rainbow (with higher values and variability in plants grown under severe water stress), but in Illpa the moderate water stress induced higher levels than in the other two treatments (Figure 6). 

### 2.6. Relationship between Leaf ABA Concentration and Stomatal Closure under Water Stress

**Figure 7:**
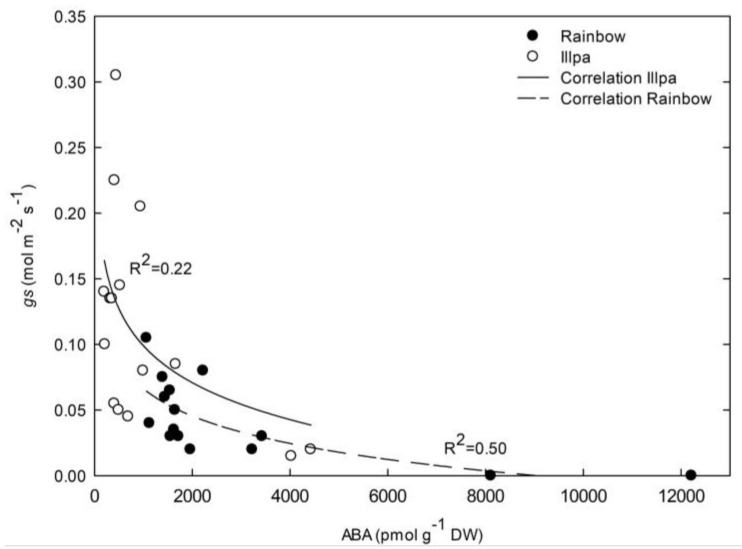
Response of stomatal conductance (gs, mol m^−2^ s^−1^) to leaf ABA concentration (pmol g^−1^ DW) in two quinoa cultivars (Rainbow (closed circles) and Illpa (open circles)) grown under irrigation regimes of 100% substrate holding full capacity (FC), 50% FC or 20% FC.

When data from all three treatments were plotted together, negative, logarithmic relationships between leaf ABA concentrations and stomatal conductances were found, showing significant R^2^ values of 0.50 (*P* = 0.0016) and 0.22 (*P* = 0.0392) in Rainbow and Illpa, respectively (Figure 7). These numbers indicate that 50% and 78% of the stomatal closure variability was not due to leaf ABA in Rainbow and Illpa, respectively. When data from the two varieties were plotted as a single data set (without differentiating between varieties), an R^2^ value of 0.43 (*P* = 4.3 10^−5^) was found (not shown). The data seem to indicate that quinoa closes stomata via both ABA-dependent and ABA-independent mechanisms.

## 3. Discussion

When water stress is applied during the grain-filling period in cereals, it commonly reduces the grain yield, grain number per plant and individual grain weight [16]. In the Mediterranean area, low soil water availabilities are frequent, coinciding with the cereal grain-filling period leading to low-yield harvests. However, in our study the quinoa cultivars from two different geographical origins (coastal and altiplano zones) did not show significant reduction in grain yield among treatments, except in 1000-grain weight for the altiplano cultivar (Illpa) (Table 1). Similar to our results, a recent greenhouse study carried out in quinoa showed that water deficit did not affect seed yield [23]. The low effect of water stress on yield could be due to an enhanced remobilization of pre-stored reserves driven towards grain filling, as reported in rice [19] and pigeonpea [24]. In contrast, studies in barley and wheat plants have found reduced grain yield when the water stress was applied post anthesis [18,25]. With regard to quinoa seed quality in both of the cultivars, most of the parameters were not significantly affected by water deficit. This suggests a possible effect of remobilization of reserves to grain, as mentioned above.

The absence of a significant reduction in grain yield in the two quinoa cultivars is due to a number of morphological, physiological and metabolic strategies that plants use to combat water stress. At a morphological level, we observed a strong enhancement in root biomass (Figure 2). Previous studies have indicated that quinoa plants have high plasticity and tissue elasticity in their roots to capture water [26], in addition to an ability to increase the growth, the depth and the density of the root system [9,10,27] to access water deep in the soil. This trait is considered important in determining plant drought resistance [16,28]. Further growth of the root system is likely supported by the starch remobilization from leaves to roots and/or its degradation to release sugars to provide energy when photosynthesis is limited [29]. For this reason, leaf carbohydrates can decrease in source organs, as observed with the leaf starch concentration in both cultivars and soluble sugars in Rainbow (Figure 4). 

The intrinsic water use efficiency (WUE*i*) is considered an important parameter for water-scarcity adaptation [30]. In our study, no significant differences were found in WUE*i* values among treatments in Rainbow, and there were only small differences in Illpa (Figure 3E). The WUE*i* determination is based on gas exchange measurements taken at a single time [31], which cannot give reliable differences in WUE*i* [32]. In contrast, the discrimination of ^13^C (Δ^13^C) provides an integration of photosynthetic activity throughout the period that leaf tissue is synthesized, reflecting aspects of plant carbon and water relations [31]. In this study, the Δ^13^C decreased significantly in Rainbow in plants under water stress but not in Illpa. Previous studies have indicated a negative correlation between Δ^13^C and WUE [33,34]. Thus, the results in our study could indicate higher WUE in Rainbow than in Illpa under water-limited conditions, with Rainbow having greater control over stomatal closure.

Furthermore, photosynthesis is among the primary processes to be affected by drought. According to several studies in quinoa under greenhouse conditions, photosynthetic parameters decrease with water stress [9,23,35], in line with the results of *An*, *gs* and E in the present study (Figure 3A–C). However, Jacobsen et al. [9] indicated that photosynthetic parameters (*An* and *gs*) can be fairly stable 10 days after application of water deficit treatments, even when stress increases. The effects can be caused by (i) diffusion limitations through stomatal closure and (ii) biochemical limitations, mainly to Rubisco, that affect the CO_2_ assimilation rate [36]. Causes of the decreased photosynthesis in Illpa could be both diffusional and biochemical, because its stomata closed (Figure 3B), its Rubisco content decreased (Figure 5) and *Ci* values were unaffected (Figure 3D), which suggests a co-dominance of stomatal and biochemical limitations, under water stress conditions. In Rainbow, the results were not so clear. Photosynthetic decreases in Rainbow were accompanied by non-significant decreases in *gs* and Rubisco content. Further, stomatal conductance may affect the sub-stomatal CO_2_ concentration (*Ci*) [11,16], and this was certainly our observation in Rainbow under water stress, which would suggest a predominance of diffusional limitations (Figure 3D). Similar results have been reported in quinoa under drought in field conditions, which indicate no direct correlations between *Ci* and *gs* [30]. 

Osmotic adjustment consists of solute and/or ion accumulation in leaves and roots aimed at maintaining tissue turgor at the cellular level. It is considered an important strategy for plant adaptation to water-limited environments [37]. Osmotic adjustment includes the accumulation of (i) compatible organic solutes, such as sugars and amino acids, and (ii) inorganic ions, both of which attract water and maintain cell turgor [37]. Here the cultivars’ responses were different with respect amino acid accumulation in the leaves (Figure 4). In Rainbow, glutamate and glutamine were the most abundant under severe water stress, whereas in Illpa it was GABA. Glutamate and glutamine are involved in the assimilation and translocation of N from source to sink organs and are also known to play a role in acclimation of plants to water stress by providing osmotic adjustment to defend against drought [38]. Such nitrogenous compounds may be derived from N assimilation or proteolysis [39]. In plants tolerant to water deficit, compatible solutes are bound to protein surfaces thus stabilizing the native protein structure, while in sensitive plants, the proteins tend to be degraded [40]. In this study, Rainbow apparently uses N assimilation to increase the amino acid concentration in its leaves, while Illpa uses both the proteolysis strategy and sugar accumulation in response to water deficit. Evidence for this comes from the lower relative Rubisco content (proteolysis strategy) and the high accumulation of soluble sugars under severe water stress in Illpa (the latter in line with Zhong et al. [39]). A study of five cultivars of quinoa plants grown under water deficit conditions reported increases in threonine and methionine, and this suggests that the adaptation strategy and the type of amino acid accumulated depends on the cultivar evaluated and its origins [41]. In addition, inorganic ions play an important role in osmotic adjustment, with K being reported in particular [37,42]. In this study, K was accumulated in Illpa, but in Rainbow Na was increased significantly under severe water stress (Figure 4). Previous studies in quinoa under water deficit have shown a marked increase in Na with slight accumulation of K [12]. Other authors have also reported the importance of Na ions in quinoa plants that have a remarkable tolerance to drought and salinity conditions [11]. 

ABA is involved in signaling during plant responses to abiotic stress, and especially under drought [43]. In this study, the leaf ABA concentration was not significantly different among treatments in Rainbow, although in Illpa the plants under moderate water stress had higher levels (Figure 6). These results indicate low effects of water stress on leaf ABA levels, in particular in Rainbow. Other studies in quinoa have reported an apparent minor role of ABA regulation in plants under stress, indicating other mechanisms of action in xylem sap, such as anti-transpirant compounds [9]. Also, Morales et al. [22] reported low expression levels of genes in the ABA biosynthesis pathway in quinoa plants under water stress, which may suggest that plants respond to drought through an ABA-independent mechanism. On the other hand, Alandia et al. [23] showed a significant increase in leaf ABA levels in quinoa under moderate water stress, in line with our results in Illpa (Figure 6). The same authors stated that ABA levels in quinoa might have a low effect on inducing stomatal closure [23]. Our results agree with this idea. When leaf ABA concentrations and stomatal conductance data from all three treatments were plotted together, negative logarithmic relationships were found (*R*^2^ = 0.50, *p* = 0.0016 in Rainbow; *R*^2^ = 0.22, *p* = 0.0392 in Illpa) (Figure 7). These numbers indicate that 50% and 78% of the stomatal closure variability was not due to the leaf ABA levels in Rainbow and Illpa, respectively. These moderate-to-medium values suggest that, in addition to ABA signaling, other causes for stomatal closure in quinoa under water stress such as hydraulic regulation through a change in turgor may play a role. It is known that quinoa under drought produces other anti-transpirant compounds than ABA [9] and face to some extent “hormonal stress”, suggesting other class of plant hormones like cytokinin and ethylene as research targets in water-stressed quinoa [22]. 

## 4. Materials and Methods

### 4.1. Plant Material and Experimental Design 

The study was carried out in two cultivars of quinoa (*Chenopodium quinoa* Willd.), Illpa (altiplano zone) and Rainbow (coastal zone) from Chile. The altiplano zone is characterized by harsh climate because of drought, frost and other adverse conditions. It is localized between 3500 and 4000 m above sea level, with 400 to 500 mm of precipitation in Peru and only 200 mm in Bolivia. The soils usually had pH 9 [44,45]. In contrast, the coastal zone is localized between 0 and 500 m above sea level, maximum high temperatures as high as 35°C are common and the soils usually are sandy and salinized [46]. 

Plants were germinated and 10-days-old seedlings were transferred to pots (5 L) with a peat:perlite:vermiculite mixture (2:2:1, in volume) as substrate and grown in a greenhouse under a 23°C/18°C (day/night) temperature regime until grain maturation of all plants (from 27 October 2017 to 21 February 2018 in Rainbow and 5 June 2018 in Illpa). Plants were watered with Hoagland nutrient solution.

Experimental design was completely randomized. Plants were irrigated with three irrigation regimes: 100% substrate holding full capacity (FC) as a control, 50% FC or moderate water stress and 20% FC or severe water stress assigned randomly to plants. Once a week control plants were irrigated with water, while water-stressed plants were always irrigated with Hoagland nutrient solution. Water stress was applied from the beginning of grain filling until harvest. This research was performed with a mixture of artificial substrates (peat:perlite:vermiculite) in pots and with plants growing in a greenhouse, using the % of reduction of applied water as a way of modulating water deficit and using stomatal conductance as a reference parameter of the plant water stress that plants were exposed. The response of quinoa under field conditions could be different.

### 4.2. Plant Growth 

When plants reached physiological maturity, 4–5 plants per treatment combination were harvested and later dried at 60 ºC in an oven for 48 h; afterwards the dry mass (DM) was determined. Grain and root samples were collected. Also, we determined thousand-grain weight in each plant. 

### 4.3. Mineral Composition Analyses

Mineral concentrations in grain and leaf samples were determined after digestion using ICP/OES (inductively coupled plasma/optical emission spectrometry, iCAP 6500 Duo, Thermo Fisher Scientific, Waltham, USA). 

C and N concentration (%) analyses were based on sample dynamic combustion, using an elemental analyzer (FlashEA1112, ThermoFinnigan) equipped with a MAS200R autosampler. The sample was weighed in a tin capsule (MX5 microbalance, Mettler-Toledo) and introduced into a quartz reactor filled with WO_3_ and copper and heated at 1020ºC. The combustion gas mixture was carried by a helium flow to a WO_3_ layer to achieve a complete quantitative oxidation, following by a reduction step in a copper layer to reduce nitrogen oxides and SO_3_ to N_2_ and SO_2_. The resulting components, N_2_, CO_2_, H_2_O and SO_2_ were separated in a chromatographic column (Porapak 2m) and detected with a thermal conductivity detector.

### 4.4. Physiological Measurements

Gas exchange measurements were carried out 7 days after treatment applications (grain filling). Healthy and fully developed leaves were used to measure leaf photosynthetic rate (*An*), stomatal conductance (*gs*), transpiration (*E*) and sub-stomatal CO_2_ concentration (C*i*). Measurements were made at a photosynthetic photon flux density (PFFD) of 1200 µmol m^−2^ s^−1^ using a LCi-SD (ADC BioScientific Ltd., UK).

Leaf samples (≈0.1 g dry weight) were also collected at harvest to measure the C isotopic composition (δ^13^C) using an elemental analyzer (EA1108; Carlo Erba Instrumentazione, Milan, Italia) coupled to an isotope ratio mass spectrometer (Delta C; Finnigan, Mat., Bremen, Germany) operating in continuous flow mode. The δ^13^C values were transformed to discrimination values (Δ) according to Farquhar et al. [33] (1989), where δ air = -8‰ in Vienna Pee Dee Belemnite (V-PDB):

$$\Delta^{13}C=\frac{\delta \;air-\delta \;plant}{1-\frac{\delta \;plant}{1000}}$$

### 4.5. Carbohydrates and Amino Acids at Grain Filling

Starch was determined in grain and leaf pellets after ethanol extraction, using the amyloglucosidase test kit (R-Biopharm AG, Darmstadt, Germany). The supernatant was used to determine glucose, fructose and sucrose concentrations using an ionic chromatographer (ICS-3000, Thermo Scientific™, USA).

Amino acids were determined in grain and leaf samples (≈0.2 g dry weight) after derivatization with a ACCQ-Fluor™ Reagent kit (Waters, USA) based in borate buffer, acetonitrile and AQC derivatizing reagent (6-aminoquinolyl-N-hydroxysuccinimidyl carbamate) using high performance liquid chromatography (HPLC).

### 4.6. Relative Rubisco Content

Lyophilized leaf tissue (≈0.2 g) was homogenized with extraction buffer (Tris-HCl 50 mM pH 8, EDTA 0.5 M, 2-mercaptoethanol 10 mM, DTT 1 M, MgSO_4_ 10 mM, cysteine 0.5 M, PVP 0.5%, PMSF 0.2 M). Samples were centrifuged (16000 *g*, 4°C, 10 min) and the supernatant was collected. Samples were mixed with loading buffer (Tris-HCl pH 6.8, glycerol 50%, 2-mercaptoethanol 5%, SDS 2.3%, blue bromophenol 0.1%) and boiled at 100°C for 5 min. Electrophoresis was carried out in acrylamide gels (12%) and samples had 10 µg total protein. The gels were analyzed with Coomasie Brilliant Blue. The signal bands were analyzed with Gene-Tools Analyzer software (Syngene, Cambridge, UK). 

### 4.7. Leaf Abscisic Acid Concentration

Extraction, purification, and quantification of abscisic acid (ABA) were carried out as described by Torres et al. [46], using a high resolution mass spectrometry (HPLC-ESI-HRMS) system, with some modifications: freeze-dried material (0.015 g) was used instead of frozen powdered material (0.1 g), and the residue obtained after the final evaporation was re-dissolved in 0.25 mL instead of 0.5 mL.

### 4.8. Statistical Analysis 

Statistical analysis was carried out with a completely randomized one-way ANOVA for each cultivar. The treatments were defined according to the three irrigation regimes applied to plants: 100% FC (control), 50% FC (moderate water stress) and 20% FC (severe water stress). Each treatment consisted of five individual plants, totaling 15 plants per cultivar. Sampling ranged from n = 3 to 10 replicates, depending whether less than the total number of plants per treatment were sampled or whether we took (up to) two measurements/samples per plant and treatment. The ANOVA was analyzed with R software (RStudio^®^ v.3.4.2, 2017; Boston-Seattle, USA), using the Tukey test for comparisons between treatments. Differences were considered significant when *P* < 0.05.

## 5. Conclusions

Despite the maintenance of production in both cultivars under water stress, according to the grain yield results, and especially the 1000-grain weight, the altiplano cultivar (Illpa) was more affected than the coastal cultivar (Rainbow). The results indicated that different mechanisms existed in the two quinoa cultivars to cope with drought and to avoid its effects on yield and grain quality. These included enhanced root growth, increased stomatal closure to reduce photosynthesis and transpiration, and lower ^13^C discrimination, but with these mechanisms being stronger in the coastal cultivar. Amino acids related to N assimilation, such as glutamine, and Na ion accumulation had effects on osmotic adjustment in the coastal cultivar, while the altiplano cultivar apparently favored osmotic adjustment through soluble sugars and K ions with a high level of protein degradation to release amino acids. Finally, drought had a low effect on ABA levels in the coastal cultivar, but it increased under moderate water stress in the altiplano cultivar. The negative logarithmic relationships in both varieties in the response of stomatal conductance to leaf ABA, which showed moderate-to-medium values, suggested that other mechanisms such as hydraulic regulation might play a role in stomatal closure in quinoa under drought.

## Figures and Tables

**Table 1:** Grain yield (g plant^−1^), thousand-grain weight (g) and grain number in the coastal (Rainbow) and altiplano (Illpa) cultivars grown under irrigation regimes of 100% substrate holding full capacity (FC), 50% FC or 20% FC. The same letters indicate no significant differences between treatments for a given cultivar (*p* < 0.05). Mean ± SE (*n* = 4–5).

Irrigation	Grain Yield (g Plant^−1^)		Thousand-Grain Weight (g)		Grain Number	
	Rainbow	Illpa	Rainbow	Illpa	Rainbow	Illpa
100% FC	15.66 ± 2.09	3.79 ± 0.92	2.07 ± 0.04	3.20 ± 0.17a	7480 ± 1278	1244 ± 360
50% FC	13.44 ± 0.83	3.30 ± 1.34	1.96 ± 0.06	2.81 ± 0.26ab	6871 ± 631	1319 ± 622
20% FC	16.78 ± 1.50	2.79 ± 1.09	2.09 ± 0.05	2.38 ± 0.17b	8142 ± 1233	1181 ± 506

## Supplementary Materials

The following are available online at https://www.mdpi.com/2223-7747/8/6/173/s1, Table S1: Grain quality parameters in two quinoa cultivars (Rainbow and Illpa) grown under full irrigation or two different levels of water deficit applied during the grain filling period, Table S2: Carbohydrates, amino acids and minerals composition in leaves of two quinoa cultivars (Rainbow and Illpa) grown under full irrigation or two different levels of water deficit applied during the grain filling period.

## References

[1] Repo-Carrasco R., Espinoza C., Jacobsen S.E. (2003). Nutritional value and use of the andean crops quinoa (*Chenopodium quinoa*) and kañiwa (*Chenopodium pallidicaule*). Food Rev. Int..

[2] Abugoch L.E. (2009). Quinoa (Chenopodium quinoa Willd.): Composition, chemistry, nutritional, and functional properties. Advances in Food and Nutrition Research.

[3] Gordillo-Bastidas E., Díaz-Rizzolo D., Roura E., Massanés T., Gomis R. (2016). Quinoa (*Chenopodium quinoa* Willd), from Nutritional Value to Potential Health Benefits: An Integrative Review. J. Nutr. Food Sci..

[4] Jarvis D.E., Ho Y.S., Lightfoot D.J., Schmöckel S.M., Li B., Borm T.J.A., Ohyanagi H., Mineta K., Michell C.T., Saber N. (2017). The genome of Chenopodium quinoa. Nature.

[5] Jacobsen S.E., Mujica A., Jensen C.R. (2003). The resistance of quinoa (*Chenopodium quinoa* Willd.) to adverse abiotic factors. Food Rev. Int..

[6] Bates B., Kundzewicz Z., Wu S., Palutikof J. (2008). El Cambio Climático y el Agua.

[7] Georgakopoulos P., Travlos I.S., Kakabouki I., Kontopoulou C.K., Pantelia A., Bilalis D.J. (2016). Climate Change and Chances for the Cultivation of New Crops. Not. Bot. Horti Agrobo.

[8] Jellen E., Maughan P., Bertero D., Munir H., Mohan S., Gupta D. (2013). Prospects for Quinoa (*Chenopodium Quinoa* Willd.) improvement through biotechnology. Biotechnology of Neglected and Underutilized Crops.

[9] Jacobsen S.E., Liu F., Jensen C.R. (2009). Does root-sourced ABA play a role for regulation of stomata under drought in quinoa (*Chenopodium quinoa* Willd.). Sci. Hortic..

[10] Jensen C., Jacobsen S.-E., Andersen M., Núñez N., Andersen S., Rasmussen L., Mogensen V. (2000). Leaf gas exchange and water relation characteristics of field quinoa (*Chenopodium quinoa* Willd.) during soil drying. Eur. J. Agron..

[11] Razzaghi F., Jacobsen S., Jensen C.R., Neumann M. (2015). Ionic and photosynthetic homeostasis in quinoa challenged by salinity and drought—mechanisms of tolerance. Funct. Plant Biol..

[12] Sun Y., Liu F., Bendevis M., Shabala S., Jacobsen S. (2014). Sensitivity of two quinoa (*Chenopodium quinoa* Willd.) varieties to progressive drought stress. J. Agron. Crop. Sci..

[13] Telahigue D.C., Ben Laila Y., Aljane F., Belhouchett K., Toumi L. (2017). Grain yield, biomass productivity and water use efficiency in quinoa (*Chenopodium quinoa* Willd.) under drought stress. J. Sci. Agric.

[14] Alvarez-Flores R., Winkel T., Degueldre D., Del Castillo C., Joffre R. (2014). Plant growth dynamics and root morphology of little-known species of Chenopodium from contrasted Andean habitats. Botany.

[15] Gomaa E.F. (2014). Studies on Some Micro-Macromorphological and Anatomical Characters of Quinoa. J. Agric. Biol. Sci..

[16] Farooq M., Wahid A., Kobayashi N., Fujita D., Basra S. (2009). Review article Plant drought stress: Effects, mechanisms and management. Agron. Sustain. Dev..

[17] Daryanto S., Wang L., Jacinthe P.A. (2017). Global synthesis of drought effects on cereal, legume, tuber and root crops production: A review. Agric. Water Manag..

[18] Estrada-Campuzano G., Miralles D.J., Slafer G.A. (2008). Genotypic variability and response to water stress of pre-and post-anthesis phases in triticale. Eur. J. Agron..

[19] Yang J., Zhang J., Wang Z., Zhu Q., Wang W. (2001). Remobilization of carbon reserves in response to water deficit during grain filling of rice. Field Crop. Res..

[20] Flagella Z., Giuliani M.M., Giuzio L., Volpi C., Masci S. (2010). Influence of water deficit on durum wheat storage protein composition and technological quality. Eur. J. Agron..

[21] Fischer S., Wilckens R., Jara J., Aranda M., Valdivia W., Bustamente L., Graf F., Obal I. (2017). Protein and antioxidant composition of quinoa (*Chenopodium quinoa* Willd.) sprout from seeds submitted to water stress, salinity and light conditions. Ind. Crop. Prod..

[22] Morales A., Zurita-Silva A., Maldonado J., Silva H. (2017). Transcriptional Responses of Chilean Quinoa (*Chenopodium quinoa* Willd.) Under Water Deficit Conditions Uncovers ABA-Independent Expression Patterns. Front. Plant Sci..

[23] Alandia G., Jacobsen S., Kyvsgaard N.C., Condori B., Liu F. (2016). Nitrogen Sustains Seed Yield of Quinoa Under Intermediate Drought. J. Agron. Crop. Sci..

[24] Subbarao G.V., Nam N.H., Chauhan Y.S., Johansen C. (2000). Osmotic adjustment, water relations and carbohydrate remobilization in pigeonpea under water deficits. J. Plant Physiol..

[25] Samarah N. (2005). Effects of drought stress on growth and yield of barley. Agron. Sustain. Dev..

[26] Vacher J.J. (1998). Responses of two main Andean crops, quinoa (*Chenopodium quinoa* Willd) and papa amarga (*Solanum juzepczukii* Buk.) to drought on the Bolivian Altiplano: Significance of local adaptation. Agric. Ecosyst. Environ..

[27] Jacobsen S.E., Jensen C.R., Liu F. (2013). Improving Crop Production in the Arid Mediterranean Climate. Field Crop. Res..

[28] Kavar T., Maras M., Kidrič M., Šuštar-Vozlič J., Meglič V. (2008). Identification of genes involved in the response of leaves of *Phaseolus vulgaris* to drought stress. Mol. Breed..

[29] Thalmann M., Santelia D. (2017). Starch as a determinant of plant fitness under abiotic stress. New Phytol..

[30] González J.A., Bruno M., Valoy M., Prado F.E. (2011). Genotypic Variation of Gas Exchange Parameters and Leaf Stable Carbon and Nitrogen Isotopes in Ten Quinoa Cultivars Grown under Drought. J. Agron. Crop. Sci..

[31] Dawson T.E., Mambelli S., Plamboeck A.H., Templer P.H., Tu K.P. (2002). Stable Isotopes in Plant Ecology. Annu. Rev. Ecol. Syst..

[32] Tambussi E.A., Bort J., Araus J.L. (2007). Water use efficiency in C_3_ cereals under Mediterranean conditions: A review of physiological aspects. Ann. Appl. Biol..

[33] Farquhar G.D., Ehleringer J.R., Hubick K.T. (1989). Discrimination and Photosynthesis. Annu. Rev. Plant Physiol. Plant Mol. Biol..

[34] Zhang C., Zhang J., Zhao B., Zhang H., Huang P. (2009). Stable Isotope Studies of Crop Carbon and Water Relations: A Review. Agric. Sci. China.

[35] Yang A., Akhtar S.S., Amjad M., Iqbal S., Jacobsen S.E. (2016). Growth and Physiological Responses of Quinoa to Drought and Temperature Stress. J. Agron. Crop. Sci..

[36] Flexas J., Díaz-Espejo A., Conesa M.A., Coopman R.E., Douthe C., Gago J., Gallé A., Medrano H., Ribas-Carbo M., Tomás M. (2016). Mesophyll conductance to CO_2_ and Rubisco as targets for improving intrinsic water use efficiency in C_3_ plants. Plant Cell Environ..

[37] Turner N.C. (2018). Turgor maintenance by osmotic adjustment: 40 years of progress. J. Exp. Bot..

[38] Tegeder M., Masclaux-Daubresse C. (2018). Source and sink mechanisms of nitrogen transport and use. New Phytol..

[39] Zhong C., Cao X., Bai Z., Zhang J., Zhu L., Huang J., Jin Q. (2018). Nitrogen metabolism correlates with the acclimation of photosynthesis to short-term water stress in rice (Oryza sativa L.). Plant Physiol. Biochem..

[40] Hoekstra F.A., Golovina E.A., Buitink J. (2001). Mechanisms of plant desiccation tolerance. Trends Plant Sci..

[41] Al-Naggar A., El-Salam R., Badran A., El-Moghazi M. (2017). Drought tolerance of Five Quinoa (*Chenopodium quinoa* Willd.) Genotypes and Its Association with Other Traits under Moderate and Severe Drought Stress. Asian J. Adv. Agric. Res..

[42] Blum A. (2017). Osmotic adjustment is a prime drought stress adaptive engine in support of plant production. Plant Cell Environ..

[43] Laloum T., Martín G., Duque P. (2018). Alternative Splicing Control of Abiotic Stress Responses. Trends Plant Sci..

[44] Jacobsen S. (2011). The situation for Quinoa and its production in southern Bolivia: From economic success to environmental disaster. J. Agron. Crop. Sci..

[45] Gomez L., Aguilar E., Universdad Nacional Agraria La Molina (2016). Guía de Cultivo de la Quinua.

[46] Torres N., Goicoechea N., Zamarreño A.M., Antolín M.C. (2018). Mycorrhizal symbiosis affects ABA metabolism during berry ripening in *Vitis vinifera* L. cv. Tempranillo grown under climate change scenarios. Plant Sci..

