# Supplementary material for: Effect of Water Stress during Grain Filling on Yield, Quality and Physiological Traits of Illpa and Rainbow Quinoa (Chenopodium quinoa Willd.) Cultivars
Source: Plants (Basel). 2019 Jun 14;8(6):173. doi: 10.3390/plants8060173 (PMC6631622; doi:10.3390/plants8060173)
Supplement: Supplementary file 1 [file plants-08-00173-s001.pdf]

plants MDPI

Table S1. Grain quality parameters in two quinoa cultivars (Rainbow and Illpa) grown under irrigation regimes of 100% substrate holding full capacity (FC), 50% FC or 20% FC. Parameters with different letters indicate significant differences among treatments for a given cultivar (*P<0.05*). Mean ± SE (n = 4-5).

Grain quality parameters	Rainbow			Illpa		
	**100% FC**	**50% FC**	**20% FC**	**100% FC**	**50% FC**	**20% FC**
**C (%)**	43.34 ± 0.18	43.91 ± 0.05	43.49 ± 0.18	40.43 ± 0.11	40.39 ± 0.08	40.92 ± 0.15
**N (%)**	3.12 ± 0.09	3.03 ± 0.05	3.16 ± 0.02	2.81 ± 0.12	2.75 ± 0.09	2.76 ± 0.04
**Amino acids - μg g^{-1} DW**						
	**100% FC**	**50% FC**	**20% FC**	**100% FC**	**50% FC**	**20% FC**
**Aspartic acid**	67.27 ± 7.36	79.95 ± 2.42	63.54 ± 5.85	91.00 ± 9.04	73.05 ± 5.54	86.43 ± 6.20
**Glutamic acid**	104.70 ± 20.46	134.84 ± 6.34	91.77 ± 18.47	108.00 ± 17.94	85.97 ± 11.84	90.44 ± 9.23
**Serine**	23.42 ± 0.44	26.71 ± 1.05	23.95 ± 1.05	43.89 ± 6.91	34.36 ± 3.91	32.15 ± 1.75
**Glycine**	18.51 ± 1.20	19.71 ± 0.76	17.44 ± 1.07	28.60 ± 3.50	24.34 ± 1.60	25.16 ± 0.76
**Glutamine**	**48.07 ± 2.66ab**	**54.43 ± 0.87a**	**44.55 ± 1.15b**	70.83 ± 6.00	57.39 ± 1.90	64.17 ± 5.30
**Histidine**	37.43 ± 2.76	38.77 ± 0.47	34.72 ± 1.21	51.20 ± 3.52	44.92 ± 1.60	50.17 ± 5.36
**Threonine**	43.89 ± 3.59	44.62 ± 1.11	43.16 ± 10.10	45.31 ± 2.80	41.16 ± 0.71	47.08 ± 4.61
**Alanine**	216.52 ± 27.76	227.20 ± 8.33	188.78 ± 22.88	462.81 ± 113.96	340.28 ± 56.92	354.54 ± 45.12
**Arginine**	49.23 ± 3.58	51.37 ± 0.67	45.88 ± 2.02	78.64 ± 11.00	67.57 ± 5.12	73.55 ± 7.55
**GABA**	22.36 ± 0.63	23.68 ± 1.13	23.65 ± 1.16	39.15 ± 4.36	37.19 ± 5.33	41.51 ± 4.66
**Proline**	38.20 ± 4.54	38.17 ± 1.22	32.30 ± 2.58	55.24 ± 7.99	43.49 ± 5.81	53.02 ± 8.91
**Tyrosine**	59.97 ± 2.31	61.93 ± 0.31	58.51 ± 1.57	76.55 ± 2.39	71.84 ± 2.57	73.33 ± 2.14
**Valine**	23.20 ± 3.09	25.90 ± 0.90	19.46 ± 2.31	32.51 ± 5.76	26.45 ± 4.21	26.30 ± 2.90
**Methionine**	29.71 ± 0.61	31. ± 0.0004	0.03 ± 0.001	50.26 ± 11.10	37.10 ± 7.49	37.68 ± 0.89
**Isoleucine**	0.035 ± 0.003	31.76 ± 0.36	30.08 ± 0.66	40.42 ± 2.56	36.74 ± 1.13	38.82 ± 1.34
**Leucine**	32.25 ± 2.02	32.31 ± 0.42	29.38 ± 1.35	39.83 ± 2.63	36.19 ± 1.30	38.85 ± 1.52
**Lysine**	2.63 ± 0.81	3.24 ± 0.42	1.83 ± 0.69	11.02 ± 4.84	5.19 ± 3.07	3.68 ± 0.96
**Phenilalanine**	46.62 ± 2.29	47.52 ± 0.92	43.84 ± 1.79	55.44 ± 1.29	53.00 ± 1.39	55.69 ± 1.30
**Total amino acids**	1017.18 ± 100.65	1094.84 ± 28.51	922.25 ± 81.07	1350.30 ± 208.58	1050.55 ± 122.28	1112.47 ± 110.22
**Carbohydrates - g 100 g^{-1} DW**						
	**100% FC**	**50% FC**	**20% FC**	**100% FC**	**50% FC**	**20% FC**
**Starch**	**20.44 ± 2.239ab**	**23.42 ± 0.504a**	**17.61 ± 0.449b**	41.13 ± 3.521a	37.97 ± 4.237a	33.57 ± 3.134a
**Glucose**	0.185 ± 0.017	0.166 ± 0.014	0.15 ± 0.012	0.107 ± 0.012	0.145 ± 0.017	0.158 ± 0.038
**Fructose**	-	-	-	0.037 ± 0.007	0.053 ± 0.012	0.104 ± 0.037
**Sucrose**	3.078 ± 0.05	2.940 ± 0.03	2.897 ± 0.07	2.287 ± 0.049	2.196 ± 0.1	2.057 ± 0.072
**Minerals - mg 100 g^{-1} DW**						
	**100% FC**	**50% FC**	**20% FC**	**100% FC**	**50% FC**	**20% FC**
**Ca**	44.822 ± 3.8	43.544 ± 2.8	37.895 ± 2.8	65.245 ± 2.32	63.979 ± 8.13	62.68 ± 6.32
**Mg**	225.009 ± 10.5	219.046 ± 4.9	206.933 ± 8.1	240.914 ± 5.95	223.202 ± 18.28	243.33 ± 10.28
**Na**	**1.319 ± 0.2a**	**0.498 ± 0.1b**	**0.386 ± 0.1b**	9.804 ± 1.11	6.986 ± 0.47	8.095 ± 1.33
**K**	1273.787 ± 64.1	1254.335 ± 47	1196.402 ± 76.9	1237.737 ± 42.57	1138.68 ± 10.61	1277.975 ± 55.86
**P**	492.201 ± 24.9	465.857 ± 9.7	445.705 ± 16.6	443.302 ± 16.36	387.81 ± 35.43	400.051 ± 21.13
**Fe**	7.239 ± 0.653	6.319 ± 0.339	5.795 ± 0.225	14.948 ± 2.32	9.665 ± 1.86	8.374 ± 0.9

**Cu**	0.429 ± 0.037	0.427 ± 0.035	0.353 ± 0.017	0.818 ± 0.06	0.618 ± 0.07	0.613 ± 0.02
**Zn**	5.166 ± 1.043	4.254 ± 0.156	3.656 ± 0.2	5.623 ± 0.42	4.493 ± 0.45	5.104 ± 0.32
**Mn**	3.336 ± 0.178	3.268 ± 0.072	3.08 ± 0.123	3.346 ± 0.2	2.893 ± 0.24	2.942 ± 0.15
**B**	**0.976 ± 0.06a**	**0.871 ± 0.03ab**	**0.757 ± 0.04b**	0.964 ± 0.23	0.62 ± 0.25	0.42 ± 0.17
**Si**	**1.687 ± 0.32a**	**0.684 ± 0.04b**	**0.752 ± 0.12b**	18.669 ± 3.02	21.918 ± 8.08	12.116 ± 3.83
**S**	147.8 ± 8.34	139.78 ± 2.78	132.93 ± 5.02	136.522 ± 3.37	122.936 ± 9.61	126.62 ± 5.55
**Total minerals**	2148 ± 114	2141 ± 64.18	2036 ± 109	2185 ± 49.41	2095 ± 136.10	2159 ± 42.51

Table S2. Carbohydrates, amino acids and minerals composition in leaves of two quinoa cultivars (Rainbow and Illpa) grown under irrigation regimes of 100% substrate holding full capacity (FC), 50% FC or 20% FC. Parameters with different letters indicate significant differences among treatments for a given cultivar (*P<0.05*). Mean ± SE (n = 8-10).

	**Rainbow**			**Illpa**		
	**100% FC**	**50% FC**	**20% FC**	**100% FC**	**50% FC**	**20% FC**
**C (%)**	33.11 ± 0.62	33.87 ± 0.24	32.27 ± 0.29	33.58 ± 0.78	34.41 ± 0.78	33.10 ± 0.73
**N (%)**	3.69 ± 0.14	3.45 ± 0.07	3.48 ± 0.12	4.08 ± 0.22	4.45 ± 0.17	4.46 ± 0.20
**Carbohydrates**						
**Starch (g 100 g^{-1} DW)**	**2.22 ± 0.29 a**	**2.81 ± 0.27 a**	**0.57 ± 0.11 b**	2.43 ± 0.21	2.10 ± 0.17	1.65 ± 0.33
**Glucose (µmol g^{-1} DW)**	**114.54 ± 18.77 a**	**88.70 ± 17.79 ab**	**50.27 ± 6.34 b**	**25.18 ± 6.90 b**	**20.28 ± 3.27 b**	**137.95 ± 15.92 a**
**Fructose (µmol g^{-1} DW)**	**43.57 ± 6.60 a**	**31.90 ± 5.22 ab**	**23.57 ± 4.05 b**	**11.82 ± 3.11 b**	**12.03 ±1.91 b**	**105.00 ± 8.94 a**
**Sacarose (µmol g^{-1} DW)**	**48.68 ± 8.25 a**	**58.61 ± 7.08 a**	**29.75 ± 4.18 b**	**10.55 ± 0.93 b**	**9.53 ± 0.74 b**	**43.49 ± 7.70 a**
**Amino acids (µg g^{-1} DW)**						
**Asp**	284.34 ± 41.22	330.50 ± 68.24	419.92 ± 49.50	222.08 ± 43.86	285.72 ± 47.09	167.15 ± 46.44
**Ala**	**2396.04 ± 400.92 ab**	**1546 ± 287.16 b**	**3451.48 ± 609.00 a**	**998.56 ± 244.77 a**	**1631.01 ± 316.96 a**	**1362.46 ± 303.56 a**
**Arg**	295.83 ± 50.25	232.51 ± 35.50	349.26 ± 49.62	**94.66 ± 19.83 b**	**184.09 ± 30.85 a**	**160.85 ± 23.05 ab**
**Asn**	204.54 ± 74.39	82.36 ± 26.15	315.57 ± 87.21	126.36 ± 38.56	165.63 ± 54.87	149.59 ± 54.61
**GABA**	278.18 ± 46.15	197.82 ± 30.14	187.73 ± 58.21	77.26 ± 12.01	167.30 ± 20.03	159.16 ± 32.32
**Gln**	**254.11 ± 39.10 b**	**268.16 ± 45.06 b**	**916.42 ± 107.06 a**	242.74 ± 79.66	166.33 ± 40.97	156.43 ± 40.28
**Glu**	**645.95 ± 143.36 b**	**884.41 ± 202.49 b**	**1499.2 ± 107.27 a**	355.99 ± 88.08	474.67 ± 96.00	294.40 ± 82.05
**Gly**	**69.45 ± 17.04 b**	**65.00 ± 11.78 b**	**113.99 ± 13.68 a**	33.71 ± 10.18	52.20 ± 11.20	44.49 ± 7.84
**His**	73.89 ± 10.97	49.65 ± 6.82	75.14 ± 6.74	42.37 ± 2.52	46.47 ± 3.46	45.43 ± 2.80
**Ile**	99.04 ± 17.19	103.00 ± 16.98	125.67 ± 11.59	51.59 ± 5.36	54.87 ± 5.69	67.62 ± 6.78
**Leu**	201.01 ± 116.60	76.80 ± 10.69	95.50 ± 12.89	52.31 ± 5.73	64.20 ± 7.34	76.13 ± 10.23
**Lys**	54.92 ± 12.09	44.60 ± 11.51	133.03 ± 44.20	12.05 ± 3.41	93.90 ± 72.46	53.78 ± 17.10
**Met**	48.96 ± 5.16	45.46 ± 2.27	42.53 ± 1.48	43.18 ± 2.33	46.19 ± 2.61	49.53 ± 3.53
**Phe**	**169.11 ± 31.17 ab**	**117.93 ± 16.40 b**	**226.25 ± 38.98 a**	78.97 ± 9.55	82.68 ± 8.11	107.19 ± 18.06
**Pro**	**259.67 ± 27.08 b**	**234.35 ± 26.25 b**	**395.89 ± 33.58 a**	68.14 ± 16.62	120.07 ± 22.88	107.15 ± 31.48
**Ser**	**133.79 ± 29.56 b**	**142.32 ± 29.46 b**	**250.51 ± 22.44 a**	66.40 ± 10.98	92.12 ± 14.07	87.19 ± 16.22
**Thr**	**171.47 ± 27.02 a**	**149.18 ± 26.21 ab**	**110.87 ± 8.47 b**	-	-	-
**Tyr**	**131.33 ± 19.96 ab**	**127.77 ± 13.61 b**	**166.95 ± 13.25 a**	82.73 ± 4.74	89.00 ± 5.16	101.26 ± 9.30
**Val**	**76.05 ± 14.31 b**	**82.21 ± 15.65 b**	**168.99 ± 15.18 a**	39.70 ± 7.29	44.86 ± 6.50	56.80 ± 9.03
**Total amino acids**	7206.13 ± 1228.72	5400.50 ± 926.40	8669.73 ± 950.99	2386.84 ± 463.79	3768.63 ± 877.86	3576.16 ± 690.09
**Minerals (mg 100 g^{-1} DW)**						
**Al**	**1.84 ± 0.20 b**	**1.41 ± 0.05 b**	**2.57 ± 0.27 a**	**5.89 ± 0.60 ab**	**6.25 ± 0.45 a**	**4.39 ± 0.30 b**

**B**	2.63 ± 0.26	2.79 ± 0.47	2.13 ± 0.36	2.04 ± 0.27	1.49 ± 0.26	1.85 ± 0.34
**Ca**	**1229.25 ± 82.89ab**	**1170.69 ± 35.73b**	**1505.38 ± 106.57a**	1119.9 ± 103.45	1068.23 ± 65.14	1060.60 ± 82.00
**Cu**	0.40 ± 0.02	0.40 ± 0.02	0.40 ± 0.02	0.43 ± 0.02	0.44 ± 0.02	0.43 ± 0.02
**Fe**	5.83 ± 0.34	4.98 ± 0.25	5.72 ± 0.36	7.36 ± 0.76	5.99 ± 0.429	6.00 ± 0.58
**K**	**7091.13 ± 232.74 b**	**6963.53 ± 186.89 b**	**8207.76 ± 282.33 a**	**8134.31 ± 913.30 a**	**9168.78 ± 641.82 ab**	**10714.06 ± 458.16 b**
**Mg**	1725.59 ± 96.14	1670.59 ± 53.59	2185.59 ± 183.05	1608.13 ± 124.01	1597.61 ± 137.33	1627.94 ± 87.15
**Mn**	**10.45 ± 0.47 b**	**10.05 ± 0.24 b**	**12.69 ± 0.98 a**	11.99 ± 0.78	12.11 ± 0.63	11.67 ± 0.60
**Na**	**21.08± 3.05 b**	**28.08 ± 4.28ab**	**38.99 ± 7.14 a**	4.50 ± 1.06	4.31 ± 0.62	5.65 ± 1.20
**P**	661.79 ± 51.74	673.91 ± 33.83	755.57 ± 55.11	420.50 ± 66.30	412.60 ± 43.67	380.48 ± 24.98
**S**	215.84 ± 14.11	199.91 ± 11.03	219.70 ± 12.58	345.69 ± 25.88	305.84 ± 17.35	361.18 ± 24.57
**Si**	**42.35 ± 2.88 b**	**43.46 ± 3.16 b**	**52.96 ± 5.97 a**	27.66 ± 3.75	27.12 ± 2.33	28.61 ± 2.22
**Zn**	1.41 ± 0.09	1.82 ± 0.16	1.46 ± 0.11	2.18 ± 0.23	2.28 ± 0.21	2.05 ± 0.12
